# β2-Adrenergic Receptors Increase Cardiac Fibroblast Proliferation Through the Gαs/ERK1/2-Dependent Secretion of Interleukin-6

**DOI:** 10.3390/ijms21228507

**Published:** 2020-11-12

**Authors:** Miles A. Tanner, Toby P. Thomas, Charles A. Maitz, Laurel A. Grisanti

**Affiliations:** 1 Department of Biomedical Sciences, College of Veterinary Medicine, University of Missouri, Columbia, MO 65211, USA; tannerm@missouri.edu (M.A.T.); tobythomas@mail.missoui.edu (T.P.T.); 2 Department of Veterinary Medicine and Surgery, College of Veterinary Medicine, University of Missouri, Columbia, MO 65211, USA; maitzc@missouri.edu

**Keywords:** β-adrenergic receptor, fibroblast, interleukin-6, cardiovascular disease, G protein

## Abstract

Fibroblasts are an important resident cell population in the heart involved in maintaining homeostasis and structure during normal conditions. They are also crucial in disease states for sensing signals and initiating the appropriate repair responses to maintain the structural integrity of the heart. This sentinel role of cardiac fibroblasts occurs, in part, through their ability to secrete cytokines. β-adrenergic receptors (βAR) are also critical regulators of cardiac function in the normal and diseased state and a major therapeutic target clinically. βAR are known to influence cytokine secretion in various cell types and they have been shown to be involved in cytokine production in the heart, but their role in regulating cytokine production in cardiac fibroblasts is not well understood. Thus, we hypothesized that βAR activation on cardiac fibroblasts modulates cytokine production to influence fibroblast function. Using primary fibroblast cultures from neonatal rats and adult mice, increased interleukin (IL)-6 expression and secretion occurred following β2AR activation. The use of pharmacological inhibitors and genetic manipulations showed that IL-6 elevations occurred through the Gαs-mediated activation of ERK1/2 and resulted in increased fibroblast proliferation. In vivo, a lack of β2AR resulted in increased infarct size following myocardial infarction and impaired wound closure in a murine dermal wound healing assay. These findings identify an important role for β2AR in regulating fibroblast proliferation through Gαs/ERK1/2-dependent alterations in IL-6 and may lead to the development of improved heart failure therapies through targeting fibrotic function of β2AR.

## 1. Introduction

Cardiac fibroblasts are a key component of the natural resident cell population in the heart. These cells are critical during homeostasis as well as in pathological cardiac states. So called “sentinel” cells, fibroblasts sense chemical, mechanical and electrical signals in the heart and initiate the proper cascade [1,2,3,4]. Cardiac fibroblasts mitigate extracellular matrix (ECM) degradation and formation through the release of regulatory proteins, controlling the flux of collagen and directly synthesizing structural/ECM proteins [5]. The release of stress signals associated with cell or tissue damage is sensed by cardiac fibroblasts and triggers an activated state [6]. In various models of heart failure, fibroblasts respond to hemodynamic or ischemic stressors by proliferating to allow for the necessary remodeling and repair [7,8,9,10]. In this state, fibroblasts become activated to myofibroblasts and adopt a new phenotypic profile. Myofibroblasts secrete ECM proteins and express contractile proteins to obtain contractile function to further contribute to the remodeling process after injury [11,12,13,14]. Proper healing and repair processes necessitate all of the aforementioned cardiac fibroblast functions in order for the presence of fibrosis or scar formation in cardiovascular disease states.

Cardiac fibroblast proliferation ensures the scaffold of the heart is maintained in the event of injury and loss of cardiomyocytes. Of the many stimuli of which fibroblasts respond to, mediators activating G protein-coupled receptors are of critical importance after injury. β-adrenergic receptors (βAR) are regulators of cardiac function in a normal and diseased state, in part, through their regulation of inflammatory responses [15,16,17,18]. In particular, regulation of the inflammatory process through activation of the β2-adrenergic receptor (β2AR) has been shown to be of importance in the context of cardiac injury [19,20]. The βAR signaling cascade is in a hyperactivated state in cardiac injury to maintain contractility and cardiac output and, therefore, inflammatory pathways stimulated by βAR are of key importance [21].

Inflammation in the heart during injury is largely mediated by pro-inflammatory cytokine release (interleukin (IL)-1β, IL-6, IL-8, CCL2, TNF-α) by immune cells and fibroblasts, which further induce myofibroblast differentiation, fibroblast proliferation and migration [22,23,24]. β2AR activation with isoproterenol has been shown to increase dermal fibroblast migration and proliferation while inducing IL-6 production, providing the impetus to study βAR activation in cardiac fibroblasts and whether this leads to the release of pro-inflammatory cytokines [25,26,27,28]. Very little is known about the role of β2AR in cardiac fibroblasts. In this study, we sought to determine whether β2AR activation, which occurs through the course of many heart failure etiologies, alters cardiac fibroblast function in terms of differentiation to a myofibroblast phenotype, proliferation, migration, and secretion of cytokines. We hypothesized that β2AR activation on cardiac fibroblasts modulates cytokine production to influence fibroblast function. By using primary murine adult and neonatal rat cardiac fibroblasts, we show that activation of the β2AR with isoproterenol induced cardiac fibroblast proliferation by augmenting IL-6 production.

## 2. Results

### 2.1. β2-Adrenergic Receptor Activation on Fibroblasts Leads to IL-6 Production

**Figure 1:**
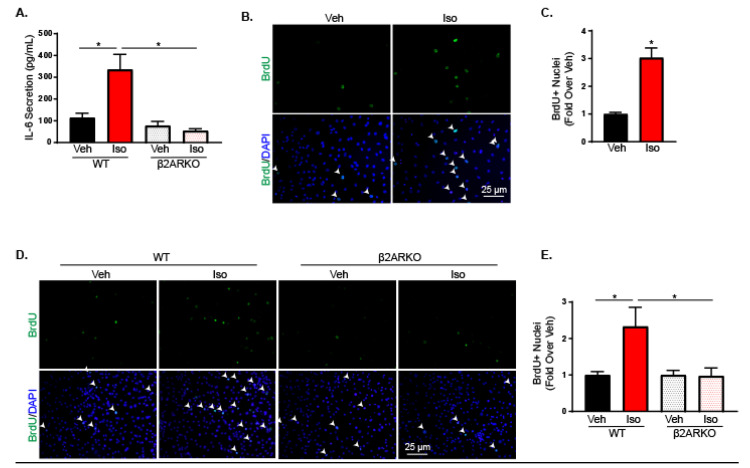
β2AR increases IL-6 production in cardiac fibroblasts. (**A**). An IL-6 ELISA was used to measure secreted IL-6 levels in media from WT or β2ARKO AMCFs. *n* = 4, one-way ANOVA, * *p* < 0.05. (**B**) Representative BrdU (green) staining alone (top) and merged with DAPI (bottom) to identify nuclei from vehicle and isoproterenol-treated RNCFs. Arrows indicate BrdU-positive nuclei. (**C**) Quantification of BrdU-positive cells from vehicle and isoproterenol-treated RNCFs. *n* = 6, *t* test, * *p* < 0.05 versus vehicle. (**D**) Representative BrdU (green) staining alone (top) or merged with DAPI (bottom) from WT and β2ARKO AMCFs treated with vehicle or isoproterenol. Arrows indicate BrdU-positive nuclei. (**E**) Quantification of BrdU-positive AMCFs from WT and β2ARKO AMCFs treated with vehicle or isoproterenol. *n* = 4, one-way ANOVA, * *p* < 0.05.

An important role of fibroblasts during pathological states is to secrete cytokines, which activate reparative responses. To determine the impact of β2AR activation on cytokine production, rat neonatal cardiac fibroblasts (RNCFs) were treated with isoproterenol and transcript levels of common cardiac cytokines were examined. Isoproterenol significantly increased IL-6 expression without altering other cytokines including TNF-α, INF-γ, IL-1β and IL-10 (Table 1). While previous studies have reported predominantly β2AR subtype expression on fibroblasts [29], which was confirmed on RNCFs (Supplemental Figure S1B) and adult mouse cardiac fibroblasts (AMCFs) (Supplemental Figure S1C), we sought to ensure IL-6 changes with isoproterenol were occurring through β2AR activation. Furthermore, mRNA levels do not always correlate with cytokine secretion and species differences can occur. Therefore, AMCFs were isolated from wild-type (WT) and β2AR knockout (KO) mice and treated with isoproterenol. Secreted IL-6 was examined by performing an IL-6 ELISA on the collected media. In WT AMCFs, isoproterenol significantly increased IL-6 secretion compared to vehicle while the increase in secretion after isoproterenol stimulation was abolished in β2ARKO cardiac fibroblasts (Figure 1A).

### 2.2. β2-Adrenergic Receptor Activation on Fibroblasts Leads to Increased Proliferation

IL-6 has been shown to influence a number of fibroblast activities including migration [30], myofibroblast differentiation [31,32,33] and proliferation [34]. To determine the functional significance of β2AR on cardiac fibroblasts, various potential outcomes were examined in RNCFs treated with isoproterenol or in WT and β2ARKO AMCFs. A scratch wound healing assay was used to investigate RNCF migration with or without isoproterenol and demonstrated no impact of βAR activation on fibroblast migration (Supplemental Figure S2A,B). Additionally, isoproterenol did not alter α-smooth muscle actin expression in the presence or absence of TGF-β, a driver of myofibroblast differentiation, indicating that β2AR is not involved in myofibroblast conversion (Supplemental Figure S2C). Furthermore, fibroblast migration (Supplemental Figure S2D,E) and α-smooth muscle actin expression were unaltered in β2ARKO AMCFs when compared with WT (Supplemental Figure S2F) and demonstrated that the lack of β2AR in adult cardiac fibroblasts had no impact on migration or myofibroblast conversion. Staining for bromodeoxyuridine (BrdU) incorporation was performed to identify replicating cells and demonstrated increased proliferation with isoproterenol administration in RNCFs (Figure 1B,C). This was also confirmed using Ki67 staining to identify mitotic cells (Supplemental Figure S3A,B). Furthermore, AMCFs isolated from WT and β2ARKO mice showed an increase in proliferation in WT AMCFs treated with isoproterenol, which was blunted in AMCFs null of β2AR signaling (Figure 1D,E).

### 2.3. Increased Fibroblast Proliferation after β2-Adrenergic Receptor Activation is PKA and ERK Dependent

**Figure 2:**
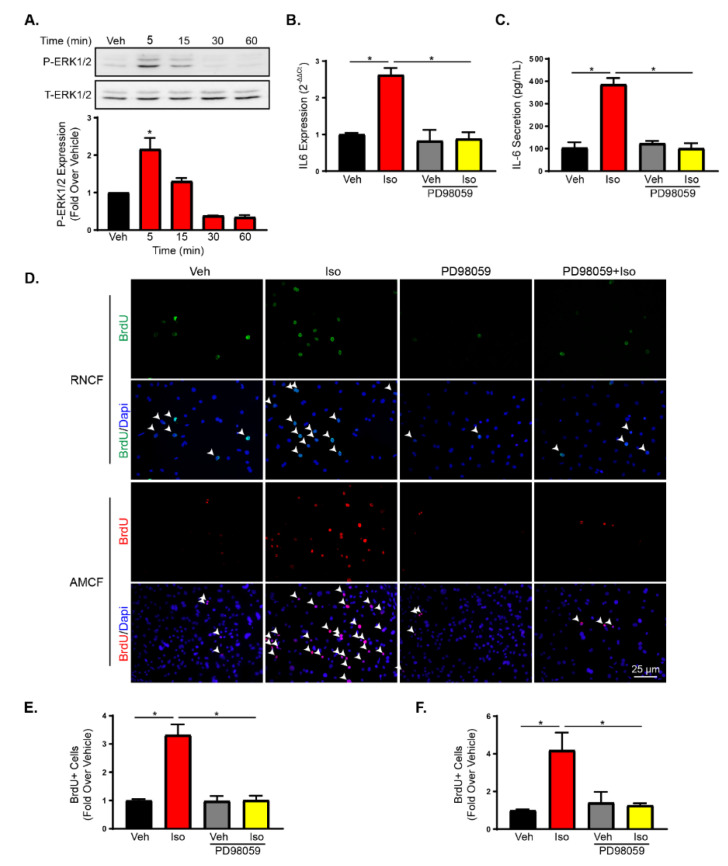
ERK1/2 activation with β2AR increases IL-6 production and proliferation in cardiac fibroblasts. (**A**) Immunoblot of phopho-ERK1/2 in the cytosol of RNCFs treated temporally with isoproterenol. Total ERK1/2 is shown as a loading control. Values are expressed as fold over vehicle treated cells. *n* = 3, one-way ANOVA, * *p* < 0.05 versus vehicle. (**B**) IL-6 transcript expression was measured by RT-qPCR in RNCFs treated with vehicle or isoproterenol in the presence or absence of PD98059. *n* = 6, one-way ANOVA, * *p* < 0.05. (**C**) IL-6 secretion was quantified using an ELISA from AMCFs from vehicle or isoproterenol-treated AMCFs with or without PD98059 pre-treatment. *n* = 6, one-way ANOVA, * *p* < 0.05. (**D**) Representative BrdU (green for RNCFs or red for AMCFs) staining alone (top) or merged with DAPI (bottom) from RNCFs or AMCFs treated with vehicle or isoproterenol in the presence or absence of PD98059. Arrows indicate BrdU-positive nuclei. (**E**) Quantification of BrdU-positive cells from RNCFs pre-incubated with PD98059 followed by vehicle or isoproterenol treatment. *n* = 6, one-way ANOVA, * *p* < 0.05. (**F**) Quantified BrdU staining from vehicle- or isoproterenol-treated AMCFs with or without PD98059 pre-treatment. *n* = 6, one-way ANOVA, * *p* < 0.05.

β2AR commonly signal through G protein-dependent or independent signal transduction mechanisms [35]. G protein-independent EGFR transactivation by β2AR has previously been linked to cardiac fibroblast proliferation [36]. To determine the impact of EGFR transactivation on IL-6 production, RNCFs were treated with isoproterenol with or without pre-treatment with the EGFR inhibitor, AG1478. Pre-incubation with AG1478 had no impact on increased IL-6 transcript expression (Supplemental Figure S4A) or proliferation (Supplemental Figure S4B,C) with isoproterenol treatment. ERK1/2 is commonly activated following β-arrestin and G protein-dependent βAR signaling [37,38]. ERK1/2 activation following isoproterenol treatment was examined in RNCFs treated temporally with isoproterenol. Phospho-ERK1/2 was elevated following βAR stimulation and peaked at 5 min post-treatment (Figure 2A). To determine the impact of ERK1/2 on IL-6 production and fibroblast proliferation, IL-6 transcript expression was examined in RNCFs treated with isoproterenol in the presence or absence of the ERK inhibitor, PD98059. Pre-treatment with PD98059 prevent isoproterenol-mediated increases in IL-6 (Figure 2B). Furthermore, PD98059 pre-treatment prevented IL-6 secretion in AMCFs (Figure 2C). To determine the impact of ERK1/2 in isoproterenol-mediated proliferation, BrdU staining was used to examine proliferation in RNCFs and AMCFs. PD98059 was also found to block isoproterenol-mediated increases in proliferation of RNCFs (Figure 2D,E) and AMCFs (Figure 2D,F).

### 2.4. β2-Adrenergic Receptor Activation Increases Fibroblast Proliferation through Gαs-Dependent Mechanisms

**Figure 3:**
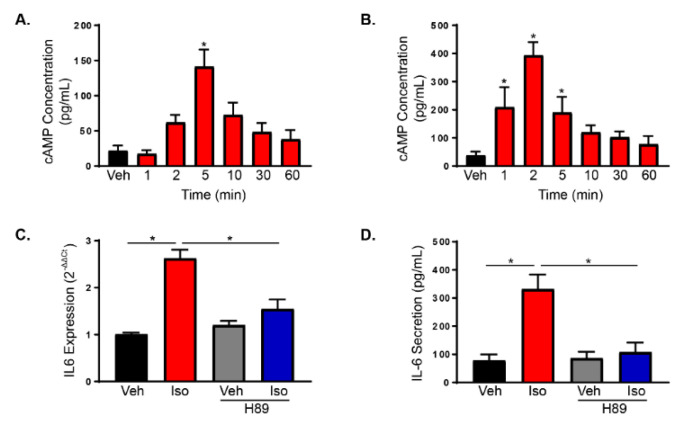
Fibroblast β2AR activates cAMP to increase IL-6 production and proliferation. (**A**) cAMP generation was quantified by ELISA in RNCFs treated temporally with isoproterenol. *n* = 3, one-way ANOVA, * *p* < 0.05 versus vehicle. (**B**) AMCFs were treated with isoproterenol over time and cAMP levels were measured by ELISA. *n* = 4, one-way ANOVA, * *p* < 0.05. (**C**) Transcript expression of IL-6 was examined by RT-qPCR in RNCFs treated with vehicle or isoproterenol with or without H89 pre-treatment. *n* = 6, one-way ANOVA, * *p* < 0.05. (**D**) An IL-6 ELISA was used to measure secreted IL-6 levels in media from AMCFs treated with vehicle or isoproterenol in the presence or absence of H89. *n* = 6, one-way ANOVA, * *p* < 0.05.

**Figure 4:**
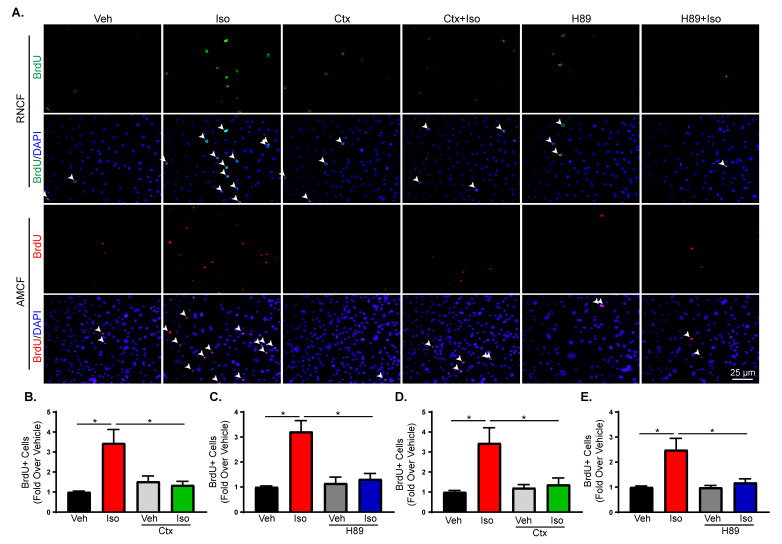
Gαs-mediated signaling alters proliferation in response to β2AR activation in cardiac fibroblasts. (**A**) Representative BrdU (green or red) staining alone (top) and merged with DAPI (bottom) to identify nuclei from vehicle and isoproterenol-treated AMCFs or RNCFs with or without cholera toxin (Ctx) or H89 pre-treatment. Arrows indicate BrdU-positive nuclei. Quantification of BrdU-positive cells from RNCFs pre-incubated with cholera toxin (**B**) or H89 (**C**) followed by vehicle or isoproterenol treatment. *n* = 6, one-way ANOVA, * *p* < 0.05. BrdU-positive AMCFs were quantified from cholera toxin (**D**) or H89 (**E**) pre-treated cells prior to vehicle or isoproterenol treatment. *n* = 6, one-way ANOVA, * *p* < 0.05.

**Figure 5:**
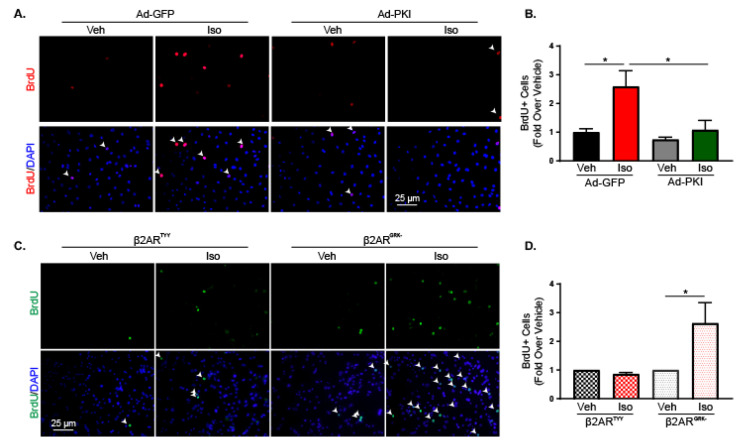
Gαs-mediated signaling alters proliferation in response to β2AR activation in cardiac fibroblasts. (**A**) Representative BrdU (red) staining alone (top) or merged with DAPI (bottom) and quantification of BrdU-positive cells (**B**) from Ad-GFP or Ad-PKI transduced RNCFs treated with vehicle or isoproterenol. Arrows indicate BrdU-positive nuclei. *n* = 5, one-way ANOVA, * *p* < 0.05. (**C**) Representative BrdU (green) staining alone (top) or merged with DAPI (bottom) and quantification of BrdU-positive nuclei (**D**) from vehicle and isoproterenol-treated β2ARKO AMCFs that were transduced with β2AR^TYY^ or β2AR^GRK-^ lentivirus. *n* = 6, one-way ANOVA, * *p* < 0.05.

Early activation of ERK1/2 has been associated with Gαs activation by β2AR in other cells types [37]. To further elucidate the mechanism of increased proliferation and IL-6 secretion after β2AR activation in cardiac fibroblasts, G protein-dependent pathways were examined. β2AR classically couple to Gαs to stimulate adenylate cyclase, elevate cAMP and activate PKA. cAMP levels were measured in RNCFs (Figure 3A) and AMCFs (Figure 3B) after isoproterenol treatment and showed activation of the canonical Gαs signaling with peaks in cAMP generation at 5 and 2 min, respectively. To confirm the involvement of G protein-dependent signaling, cholera toxin was used to inhibit adenylate cyclase and H89 was employed to inhibit PKA prior to vehicle or isoproterenol treatment in RNCFs. Pre-incubation with H89 decreased isoproterenol-mediated IL-6 expression in RNCFs (Figure 3C) and IL-6 secretion in AMCFs (Figure 3D). Furthermore, BrdU staining revealed decreased cardiac fibroblast proliferation in isoproterenol-treated RNCFs after adenylate cyclase (Figure 4A,B) or PKA (Figure 4A,C) inhibition, which also occurred in AMCFs treated with cholera toxin (Figure 4A,D) or H89 (Figure 4A,E). Furthermore RNCFs were transduced with control Ad-GFP or Ad-PKI, an endogenous inhibitor of PKA, and treated with isoproterenol. Ad-GFP-transduced cells had an increased number of BrdU-positive nuclei following isoproterenol treatment, whereas Ad-PKI prevented these changes (Figure 5A,B). To exclude the possibility of β-arrestin-mediated alterations in proliferation, RNCFs were transfected with β-arrestin 1 or 2 small interfering RNA (siRNA) and BrdU staining was used to examine proliferation. Knockdown of β-arrestin 1 or 2 had no impact on isoproterenol-induced changes in proliferation compared with control cells (Supplemental Figure S5A–C). To further confirm the role of Gαs and exclude the involvement of β-arrestin, mutant β2AR that cannot couple to Gαs but have intact β-arrestin-dependent signaling (β2AR^TYY^) [19,37,39] or β2AR that lack GRK phosphorylation sites and cannot recruit β-arrestin but have undisturbed Gαs signaling (β2AR^GRK-^) [19,37,39] were lentivirally transduced to β2ARKO AMCFs (Supplemental Figure S6C). AMCFs transduced with WT β2AR had increased BrdU staining following isoproterenol treatment, whereas RFP-expressing control cells had no response to isoproterenol, in accordance with what was previously observed in WT and β2ARKO AMCFs (Supplemental Figure S6A,B). AMCFs expressing β2AR^GRK-^ showed increased proliferation with isoproterenol administration, whereas β2AR^TYY^-transduced cells had no response (Figure 5C,D), confirming the importance of Gαs-dependent mechanisms in the proliferative response to β2AR activation.

**Figure 6:**
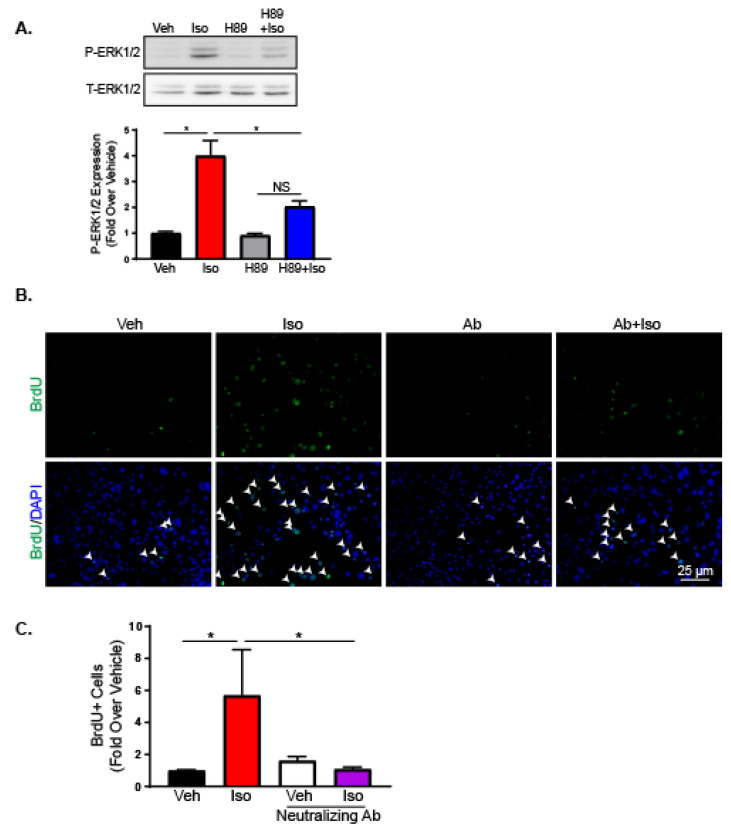
G protein-mediated signaling activates ERK1/2, leading to IL-6 production and proliferation. (**A**) Immunoblot for phospho-ERK1/2 of RNCFs pre-incubated with H89 followed by vehicle or isoproterenol treatment. Total ERK1/2 is shown as a loading control. *n* = 3, one-way ANOVA, * *p* < 0.05. NS = not significant. (**B**) Representative BrdU (green) staining alone (top) or merged with DAPI (bottom) and quantification of BrdU-positive cells (**C**) from AMCFs treated with vehicle or isoproterenol with or without pre-incubation with a neutralizing IL-6 antibody. Arrows indicate BrdU-positive nuclei. *n* = 8, one-way ANOVA, * *p* < 0.05.

Our results demonstrate the importance of Gαs-mediated and ERK1/2 signaling in increasing IL-6 expression and increasing proliferation. However, it is unclear whether Gαs-dependent mechanisms activate ERK1/2 or the two pathways operate independently. To determine the importance of G protein-dependent signaling in ERK1/2 activation, RNCFs were treated with isoproterenol with or without H89 to inhibit PKA and ERK1/2 activation was examined by Western blot. ERK1/2 phosphorylation was elevated with isoproterenol treatment in RNCFs (Figure 6A). H89 blocked isoproterenol-mediated increases in phospho-ERK1/2 levels, demonstrating the importance of G protein-dependent mechanisms in ERK1/2 activation.

In order to show elevated IL-6 is responsible for the increased proliferation observed in fibroblasts following isoproterenol treatment, IL-6 was depleted with a neutralizing antibody in AMCFs prior to vehicle or isoproterenol treatment. Proliferation was assessed through BrdU staining and showed that neutralizing IL-6 prevented the increased proliferation observed with β2AR activation (Figure 6B,C).

### 2.5. β2-Adrenergic Receptors Impact Fibroblast Function in In Vivo

**Figure 7:**
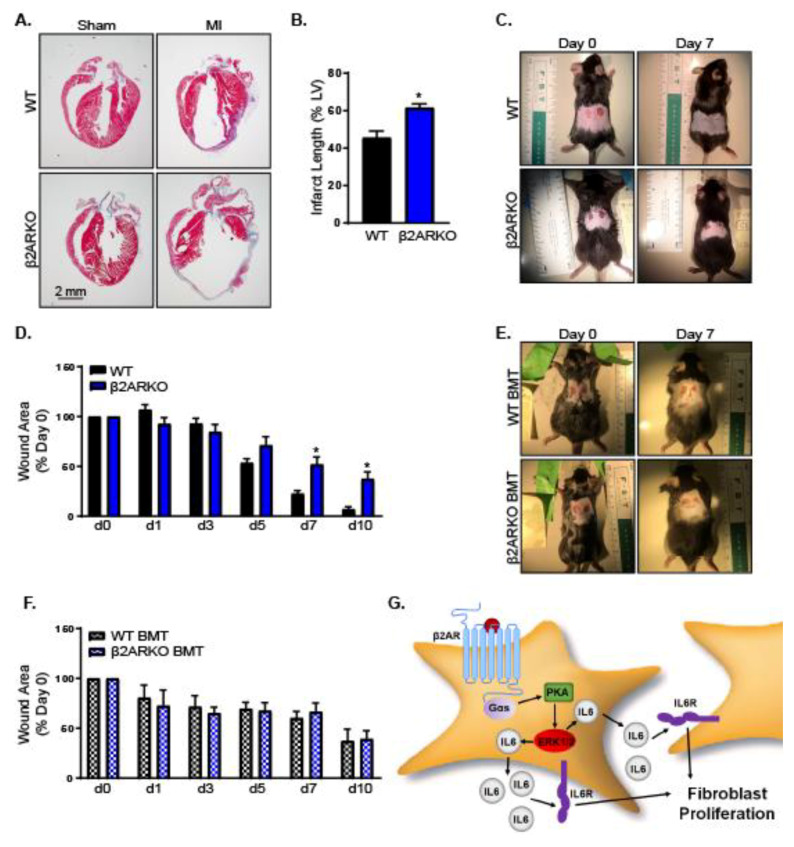
β2AR contributes to wound healing in vivo. (**A**) Representative Masson’s trichrome staining to show fibrosis (blue) and myocardium (red) in WT and β2ARKO mouse hearts following sham or myocardial infarction surgery. (**B**) Quantification of infarct length for WT and β2ARKO hearts following myocardial infarction. Infarct length was expressed as a percentage of the total left ventricle. *n* = 6 (WT) and 8 (β2ARKO), *t* test, * *p* < 0.05. (**C**) Representative images from a wound healing assay in WT and β2ARKO mice at day 0 and 7 days post-skin biopsy. (**D**) Quantification of wound healing in WT and β2ARKO. Wound area measured over time was normalized to the original wound area. *n* = 6 for WT and 7 for β2ARKO, two-way ANOVA, * *p* < 0.05 versus WT. (**E**) Representative wound healing image from WT and β2ARKO BMT mice at day 0 and day 7 following skin biopsy. (**F**) Quantification of wound healing images in WT and β2ARKO BMT mice. Wound area measured over time was normalized to the original wound area. *n* = 4, two-way ANOVA, * *p* < 0.05. (**G**) Summary schematic showing that β2AR activation on fibroblasts leads Gαs-mediated signaling, which activates ERK1/2 resulting in IL-6 generation and secretion. IL-6 can act in a paracrine or autocrine manner to increase fibroblast proliferation and influence other neighboring cells.

To determine the impact of β2AR-mediated fibroblast proliferation in vivo, WT and β2ARKO mice were subjected to sham or myocardial infarction surgery. β2ARKO mice had a larger infarct length 4 weeks following surgery, indicating impairments in fibrosis and wound healing (Figure 7A,B). Of note, β2ARKO mice had decreased survival following myocardial infarction (Supplemental Figure S7A). β2ARKO may play a role in cardiomyocyte survival and is known to play a critical role in regulating immune responses in the setting of myocardial infarction, which may be confounding factors [21,39]. To circumvent this, a dermal wound healing model was used. Wound healing in the skin occurs through many of the same mechanisms as in myocardial infarction without the influence of cardiomyocyte death and hemodynamics [40]. Increased proliferation occurred in mouse dermal fibroblasts and mouse embryonic fibroblasts treated with isoproterenol (Supplemental Figure S7B–E). These responses were abolished by IL-6 neutralizing antibody pre-treatment, suggesting that the β2AR responses observed in cardiac fibroblasts may be a universal fibroblast mechanism and applicable to other types of fibroblasts and a dermal wound healing assay may be a suitable model for what occurs in the heart. Skin biopsy punches were administered in WT and β2ARKO mice and wound closure was monitored over time. β2ARKO mice had a deceased rate of wound closure compared to WT mice, suggesting a decrease in cellular proliferation (Figure 7C,D). Importantly, this was not due to β2AR expression on immune cells, since WT mice that were irradiated to deplete hematopoietic progenitor cells and transplanted with β2ARKO bone marrow (BM) healed similarly to their WT bone marrow transplant (BMT) counterparts (Figure 7E,F).

## 3. Discussion

βAR are important regulators of cardiac function in the normal heart as well as during pathological conditions and represent a major therapeutic target for the treatment of numerous cardiovascular diseases including heart failure, arrhythmias and myocardial infarction [41,42]. The β1AR is the predominant subtype in the heart and is the primary subtype expressed on cardiomyocytes where it has been extensively studied for its role in regulating contractility, cell growth and survival [43,44,45]. The β2AR is prevalent on other cell populations in the heart including immune cells, endothelial cells and fibroblasts [39,46,47,48]. In accordance, our results demonstrate high β2AR expression on cardiac fibroblasts isolated from neonatal rats and adult mice with nearly undetectable levels of the β1AR subtype. While the influence of βAR activation has been investigated in fibroblast populations in various tissues, relatively little is known about the role of βAR in regulating fibroblast function in the heart. Thus, the present study sought to investigate the role of β2AR in regulating cardiac fibroblast function.

Fibroblasts serve as sentinels, allowing the heart to sense and respond to harmful stimuli, in part, through the secretion of cytokines [49]. βAR are known to play a role in regulating cytokine production in the heart [50,51] and other cell populations [52,53]. Therefore, the impact of βAR activation on the production of cytokines was examined with a focus on cytokines known to be derived from fibroblasts and important regulators in the heart [49]. IL-6 levels were increased in both neonatal and adult cardiac fibroblast, whereas other cytokines including IL-10 and TNF-α were unchanged. Prior studies have also implicated isoproterenol treatment in regulating IL-6 expression in cardiac fibroblasts [54,55]. These studies used pharmacological antagonist to conclude the involvement of the β2AR, which can often be non-selective depending on the dose used. Our study confirms the importance of the β2AR in regulating IL-6 production in response to isoproterenol treatment through the use of cells isolated from β2ARKO mice. Other studies have shown β2AR to regulate the production of other cytokines in addition to IL-6 in other cell types including IL-10 and TNF-α [17,56]. However, while we observed trends in increase IL-10 generation, the results were variable and not significant, while other cytokines examined were unchanged. This suggests that β2AR regulation of cytokines in fibroblast has a degree of specificity to IL-6.

In addition to cytokine production, the impact of βAR on other important fibroblast functions was examined including proliferation, migration and myofibroblast conversion, which could occur through IL-6-dependent or independent mechanisms [57]. Migration of fibroblasts treated with isoproterenol or lacking β2AR assessed using a scratch assay was unaltered compared with control cells, indicating that β2AR had no impact on cardiac fibroblast migration. In contrast to our findings, β2AR has been shown to be involved dermal fibroblast migration through src-dependent EGFR/ERK1/2 activation [58]. These studies used single-cell tracking of fibroblasts rather than a wound healing assay, which might be more sensitive at finding differences in migration than population studies. α-smooth muscle actin expression in cardiac fibroblasts treated with isoproterenol alone or in the presence of the TGF-β, a driver of myofibroblast differentiation, were unchanged. Accordingly, β2ARKO did not alter α-smooth muscle actin expression compared with WT in naïve or TGF-β-treated fibroblasts. These results suggest β2AR are no involved in the differentiation of fibroblasts. There have been some reports suggesting that βAR play an anti-fibrotic role through the induction of fibroblast autophagy [46]. However, our findings would suggest the opposite, since increased proliferation was observed in isoproterenol-treated fibroblasts, which was prevented in β2ARKO cells.

Other studies have also seen increased fibroblast proliferation with βAR agonists. βAR classically signal through Gαs-dependent mechanisms, which include the stimulation of adenylate cyclase, elevations in cAMP and activation of PKA [59]. However, alternative signaling paradigms including transactivation of EGFR, βγ signaling and β-arrestin-dependent signaling are becoming increasingly appreciated [60,61]. In order to determine the mechanism of β2AR-dependent alterations in IL-6-mediated proliferation, activation of cAMP was examined and found to be elevated with isoproterenol treatment. This is unsurprising, since βAR on cardiac fibroblasts have been found to primarily signal through Gαs, with alternative pathways playing a smaller role [62]. In certain cell types, including cardiomyocytes, β2AR can switch its G protein coupling from Gαs to Gαi [63]. This occurs through the PKA-dependent phosphorylation of β2AR, which decreases its affinity for Gαs and promotes coupling to Gαi [64]. Isoproterenol-mediated ERK1/2 phosphorylation has been shown to be pertussis toxin sensitive, indicating Gαi involvement, in some cell lines including HEK293 cells overexpressing the β2AR [64]. However, currently, there is no evidence that Gαi coupling plays a role in fibroblasts and this mechanism was not investigated in the current study. Pharmacological and genetic inhibitors of multiple stages of Gαs signaling confirmed the involvement of Gαs-mediated signaling in the alterations observed in IL-6 and proliferation. Furthermore, EGFR inhibition, β-arrestin silencing and expression of mutant receptors that are incapable of β-arrestin recruitment had no effect on isoproterenol-mediated changes in IL-6 levels or proliferation.

Early studies showed that activation of β2AR increased human cardiac fibroblast proliferation through increased cAMP [29]. While the precise mechanism was not identified, these changes occurred in an autocrine manner, since media from βAR agonist-treated fibroblasts were sufficient to induce proliferation in naïve fibroblasts. Other studies have also shown increased proliferation through PKA-dependent mechanisms and ERK1/2 activation. However, the role of ERK1/2 in the β2AR-mediated proliferation was not investigated [65]. More recent studies have shown a role for mitogen-activated protein kinases, including ERK1/2, in the fibroblast response to isoproterenol [36,65]. ERK1/2 can be activated through both G protein-dependent and independent mechanisms [37]. We found rapid (peaking at 5 min) elevations in the phosphorylation of ERK1/2 with isoproterenol stimulation. Early, transient activation of ERK1/2 has been associated with G protein-dependent mechanisms, whereas later, prolonged ERK1/2 phosphorylation is linked to β-arrestin [37]. ERK1/2 can be activated or inhibited by many signal transduction pathways including cAMP/PKA [66]. PKA can activate Rap1 in fibroblasts [67], which can either directly phosphorylate the upstream kinase MEK [68] or indirectly through activation of guanine nucleotide exchange factors [69]. Indirect Rap1 activation has also been shown to occur through B-Raf in HEK293 cells as a result of β2AR signaling [67]. PKA has also been shown to activate src upstream of ERK1/2 activation [70]. Rap1 can also be activated in some situations by cAMP in a PKA-independent manner [71,72]. While, not significantly different, there was a trend to increased ERK1/2 phosphorylation, IL-6 generation and proliferation with isoproterenol in the presence of H89, whereas ERK1/2 and Gαs inhibition completely abolished isoproterenol-mediated responses. This may indicate divergent mechanisms of ERK1/2 activation, with a PKA-independent mechanism playing a minor role in comparison with PKA. In contrast, β2AR has also been shown to increase cardiac fibroblast DNA content, an indicator or proliferation [36]. While this study also implicated ERK1/2 in the response, ERK1/2 activation was independent of G protein activation and EGFR transactivation was found to be responsible, which was found to not occur in the present study. Importantly, while these studies observe increased proliferation with β2AR activation on fibroblasts, the signal transduction mechanisms associated with these changes are inconsistent or incomplete and the causative agent of β2AR-dependent changes in proliferation were not identified.

The present study is not the first to identify a role for β2AR in increasing IL-6 levels in fibroblasts [27,54,55]. Contrary to the present study, showing involvement of Gαs-dependent ERK1/2 activation, prior studies attributed IL-6 changes to the Gαs-dependent activation of p38. Further, these studies did not identify a functional role of IL-6 generation. IL-6 is a pleiotropic cytokine that has many functions in the heart including regulation of cardiomyocyte hypertrophy and apoptosis and polarization of immune responses [57,73,74,75]. Additionally, there is abundant evidence for IL-6 influencing fibroblast functions including proliferation [76] and myofibroblast conversion [23,76]. While we did not see other functional changes in cardiac fibroblasts with β2AR activation, proliferation was increased. Neutralizing antibodies to prevent subsequent IL-6 receptor activation following IL-6 secretion confirmed the involvement of IL-6 in the proliferative effects of β2AR.

Many of the differences in the function of ERK1/2 in the same cell can be attributed to differences in subcellular localization [77,78], timing [79] or duration [80] of activation and this might contribute to how β2AR influences IL-6 and proliferation while not impacting the generation of other cytokines or alternative fibroblast functions. Once secreted, IL-6 binds to the IL-6R, leading to the activation of signal transduction processes including Jak/STAT signaling, mitogen-activated protein kinases and Akt to activate transcription factors and alter gene transcription including cell cycle genes [81]. Differences between IL-6 actions between cell types may also be attributed to different levels of regulatory proteins such as suppressors of cytokine signaling (SOCS) that work to control IL-6 signaling [82].

Increased proliferation with β2AR would suggest increased wound repair following injury. Wound healing in the heart occurs after cardiomyocyte death in instances such as myocardial infarction and follows a series of events that is analogous to other tissues. This includes hemostasis, inflammation, tissue regrowth through proliferation and maturation by cell differentiation [49]. Due to the minimal regenerative capacity of cardiomyocytes, fibrotic scar formation plays a particularly important role in the heart [83]. To investigate the impact of β2AR, a myocardial infarction model was used. Global β2AR deletion resulted in increased scar size following myocardial infarction when compared with WT mice. These findings are similar to what is seen with deletion of other fibroblast proliferation agents such as fibroblast growth factor-2, since a lack of fibroblast proliferation cumulates in impaired wound contraction and larger infarct sizes [84]. Important to note, β2ARKO mice had increased mortality following myocardial infarction, potentially due to cardiac rupture, which also results from impaired fibrotic responses, making interpretation of long-term data difficult. Cardiac studies involving β2AR also have the confounding factors of the impact of β2AR on cardiomyocytes, where they are thought to promote survival [85], and hemodynamic effects of β2AR [86]. To eliminate potential differences in injury size, a dermal injury model was used since the mechanisms involved are analogous to what occurs in the heart following injury [87]. As anticipated, β2ARKO mice had slower wound closure when compared with WT animals, suggesting impaired wound healing. This is unsurprising since our in vitro findings suggest an important of β2AR in increasing fibroblast proliferation. During injury, fibroblast β2AR are expected to be activated and likely play a physiological role in healing due to elevations in catecholamines, the endogenous ligands of β2AR, that are increased with physical or psychological stress [88]. Since β2AR are also important regulators of the immune response [15], which also contributes to wound healing, similar experiments were performed in WT and β2AR BMT mice, which lack β2AR specifically in cells of hematopoietic origin. In contrast with global β2AR deletion, β2AR BMT had wound healing that was unaltered from WT BMT, demonstrating that the reduction in wound healing observed in β2ARKO mice was not a result of changes in inflammation. However, wound area does not provide information about the quality of the scar. Immune cells are important for other aspects of fibrosis including extracellular matrix stability, which is important for scar maturation [49]. Indeed, β2AR BMT mice are prone to cardiac rupture, suggesting other impairments in the fibrotic response with immune cell β2AR deletion [39]. The global lack of β2AR or β-blocker administration is associated with decreased cardiac fibrosis [89,90,91]. Similarly, mice lacking β2AR have decreased fibrosis in response to chronic isoproterenol administration [92]. However, due to the ubiquitous expression of βAR, it is impossible to dissociate the impact of fibroblast β2AR from its role in other cell types. To date, no studies have been published examining fibroblast-specific β2AR deletion.

Taken together, we have elucidated an important pathway in cardiac fibroblasts involving the Gαs-dependent activation of ERK1/2, resulting in enhanced IL-6 production and subsequent proliferation (Figure 7E). This is anticipated to be particularly important during pathological conditions, where there is enhanced sympathetic activity, leading to activation of β2AR. Indeed, dermal wound healing assay demonstrated reduced wound closure in β2ARKO mice compared with WT showing in vivo relevance. While clinical trials involving anti-fibrotic agents in the heart have been largely disappointing to date, targeting fibrosis remains an attractive therapeutic strategy due to the known role of fibrosis in reducing ventricular compliance and furthering heart failure progression [93]. Our findings suggest that inhibiting fibroblast β2AR might be an important mechanism through which β-blockers are decreasing cardiac fibrosis and targeting β2AR may be an effective therapeutic strategy for the treatment of heart failure.

## 4. Materials and Methods

### 4.1. Experimental Animals

All animal procedures were performed with approval by the Institutional Animal Care and Use Committee at the University of Missouri protocol and ethic permission code 9996 (28 August 2020) and in accordance with the National Institutes of Health *Guidelines on the Use of Laboratory Animals*.

### 4.2. Bone Marrow Transplant

Endogenous hematopoietic stem and progenitor cells were depleted from wild-type (WT) C57BL/6J mice (8–12 wk; Jackson Laboratories, Bar Harbor, ME, USA) by lethal irradiation delivered using a linear accelerator (950 rads). Donor bone marrow (BM) was isolated from WT C57BL/6J or β2AR knockout (KO) mice and adoptively transferred (~1 × 10^7^ cells/mouse) by retro orbital injection within 24 h of irradiation as previously described [39]. BM was allowed to reconstitute for 1 month prior to experimentation. Reconstitution was confirmed at the conclusion of this study for each mouse by reverse transcription-quantitative PCR (RT-qPCR) analysis for ADRB2 expression in BM (Supplemental Figure S1A).

### 4.3. Myocardial Infarction Surgery

Myocardial infarction surgery was performed as previously described [39,94]. Mice were anesthetized with 3% isoflurane via inhalation. A small incision was made in the skin and the pectoral muscles were retracted. A small hole was made in the fourth intercostal space and the heart was popped out. The left coronary artery was sutured ~3 mm from its origin and the heart was returned to the intrathoracic space followed by closure of the muscle and skin. Animals received a single dose of buprenorphine (0.1 mg/kg) immediately after surgery.

### 4.4. Dermal Wound Healing Assay

Male WT C57BL/6, β2ARKO bred onto a C57BL/6 background, WT bone marrow transplant (BMT) and β2ARKO BMT mice, 8–12 weeks of age, were anesthetized by isoflurane (3%) and underwent biolateral 6 mm skin punch biopsies (Integra Miltex, York, PA, USA) along the dorsal midline as previously described [40]. Daily photographs of the wounds were taken and analyzed by ImageJ (National Institutes of Health, Bethesda, MD, USA) to measure area of the wound and normalized to original wound area in order to calculate the wound closure rate.

### 4.5. Primary Cell Isolations and Treatments

Adult mouse cardiac fibroblasts (AMCFs) were isolated from WT C57BL/6 and β2ARKO mice (male and female; 8–12 weeks old). Mice were euthanized and hearts were excised. Atria were removed and ventricles were digested by manual and serial enzymatic digestion with collagenase II and trypsin as previously described [95]. Myocytes were separated from fibroblasts by centrifugation at a low speed and collecting the supernatant containing fibroblasts. Cells were cultured on 2% gelatin-coated plates in DMEM containing 10% fetal bovine serum and 1% penicillin-streptomycin at 37 °C in a humidified incubator with 5% CO_2_.

Primary rat neonatal cardiac fibroblasts (RNCFs) were isolated from 1- to 2-day-old Sprague–Dawley rat pups (Charles River, Wilmington, MA, USA) by mechanical and enzymatic digestion using collagenase II and pancreatin as previously described [3,4]. Cardiac fibroblasts and cardiac myocytes were separated by pre-plating for 2 h and RNCFs were cultured on 2% gelatin-coated plates in DMEM containing 10% fetal bovine serum and 1% penicillin-streptomycin at 37 °C in a humidified incubator with 5% CO_2_.

Mouse embryonic fibroblasts were isolated from fetuses (13 days) by manual and chemical digestion using 0.25% trypsin-EDTA. Cells were plated in DMEM containing 10% fetal bovine seum and 1% penicillin-streptomycin at 37 °C in a humidified incubator with 5% CO_2_. Experiments were performed with passage 2–6 cells.

Mouse dermal fibroblasts were isolated from skin from WT C57BL/6 mice. A ~1 cm^2^ skin biopsy was incubated in 0.25% trypsin-EDTA for 30 min followed by manual digestion in fresh 0.25% trypsin-EDTA. Cells were cultured in DMEM containing 10% fetal bovine serum and 1% penicillin-streptomycin at 37 °C in a humidified incubator with 5% CO_2_.

For proliferation and IL-6 expression studies, cells underwent pre-treatments with PD980059 (0.1 μM for 10 min; Caymen Chemicals, Ann Arbor, MI, USA, cat# 10006726), H89 (0.1 μM for 10 min; Caymen Chemicals, Ann Arbor, MI, USA, cat# 10010556), AG1478 (0.1 μM for 10 min; Caymen Chemicals, Ann Arbor, MI, USA, cat# 10010244), mouse IL-6 neutralizing antibody (1 μL/mL for 1 h; R&D Systems cat# MAB406-SP1) or cholera toxin (100 ng/mL for 16 h; Sigma Aldrich, St. Louis, MO, USA, cat# C8052) followed by isoproterenol (0.1 μM for 24 h; Sigma Aldrich, St. Louis, MO, USA, cat# I6504).

Small interfering RNAs (siRNA) for rat β-arrestin 1 (Integrated DNA Technologies cat# 273081470), β-arrestin 2 (Integrated DNA Technologies, Coralville, IA, USA cat# 273081473) or control non-targeting siRNA (Dharmacon, Lafayette, CO, USA, cat# D-001810-03) were transfected as previously described [96]. In brief, siRNA was transfected 24 h post-seeding with Dharmafect I (2.5 µL/mL media) and the appropriate siRNA (25 nM) according to the manufacturer’s protocol.

### 4.6. Viral Transduction of Primary Fibroblasts

RNCFs were transduced with adenoviral constructs for PKI or GFP control 24 h prior to treatment. β2ARKO AMCFs were transduced with RFP control, WT β2AR, β2AR^TYY^, or β2AR^GRK-^ for 24 h in DMEM + 10% fetal bovine serum prior to isoproterenol stimulation as previously described [39].

### 4.7. Reverse Transcription-Quantitative Polymerase Chain Reaction

cDNA was synthesized from total RNA of BM, AMCF or RNCF culture using the High-Capacity cDNA Reverse Transcription Kit (Applied Biosystems, Waltham, MA, USA). RT-qPCR was performed using a PowerUP SYBR Master Mix (Applied Biosystems, Waltham, MA, USA) in triplicate for each sample using primers listed in Supplemental Table S1. All RT-qPCR data were analyzed using the Applied Biosystems Comparative CT Method (∆∆CT). Gene expression analysis was normalized to translationally controlled tumor protein (TPT1) and expressed as 2^−∆∆CT^.

### 4.8. Enzyme-Linked Immunosorbent Assay (ELISA)

Secreted IL-6 levels were detected using a mouse IL-6 ELISA kit (Invitrogen, Waltham, MA, USA, cat# 88-7064) according to the manufacturer’s instructions. The 96 well plates were coated overnight with Capture Antibody then blocked with Reagent Diluent. Plates were incubated with conditioned media collected from AMCFs after inhibitor and isoproterenol treatments. After incubating with media, incubations with Detection Antibody, Streptavidin-HRP and substrate solution followed. Absorbance was measured at 450 nm.

### 4.9. Immunofluorescence

Bromodeoxyuridine (BrdU) staining was carried out on cultured fibroblasts by replacing cell the culture medium with BrdU labeling solution for 2 h at 37 °C. Labeling solution was removed and fibroblasts were fixed with 4% paraformaldehyde, permeabilized with 0.2% Triton X-100 and treated with 2 M hydrochloric acid. Nonspecific interactions were blocked with 0.1% bovine serum albumin (BSA) and anti-BrdU antibody (R&D Systems, Minneapolis, MN, USA, cat# 87225; 10 μg/mL) was incubated with cells overnight at 4 °C. Cells were then washed with phosphate-buffered saline and Alexa-488 or Alexa-546 anti-mouse was used to detect BrdU. Nuclei were counterstained with DAPI and coverslips were mounted on glass slides using Prolong Gold Anti-Fade (Molecular Probes, Eugene, OR, USA, cat# P36961). Coverslips were visualized in a blinded manner at 20X magnification using a Nikon Eclipse microscope. The percentage of positive BrdU nuclei were counted by a blinded observer from 10 random fields/coverslip were calculated in relation to the number of DAPI-stained nuclei.

### 4.10. Masson’s Trichrome Staining

Masson’s trichrome was performed as previously [96] described and according to the manufacturer’s instructions (Fisher Scientific, Waltham, MA, USA).

### 4.11. Cyclic Adenosine Monophosphate (cAMP) Assay

AMCFs and RNCFs were seeded in 96 well plates at 80% confluency and treated temporally with 0.1 μM isoproterenol. The cAMP-Glo™ Assay kit (Promega, Madison, WI, USA) was performed according to the manufacturer’s instructions.

### 4.12. Immunoblot Analysis

AMCFs or RNCFs were homogenized as previously described [97]. Immunoblotting was performed overnight at 4 °C with diluted antibodies against α-smooth muscle actin (1:1000; Sigma-Aldrich cat# A5228), β-arrestin 1/2 (1:1000; Cell Signaling Technology, Danvers, MA, USA, cat# 4674), glyceraldehyde 3-phosphate dehydrogenase (GAPDH; 1:1000; Cell Signaling Technology, Danvers, MA, USA, cat# 2118), phospho-ERK1/2 (1:1000; Cell Signaling Technology cat# 9101) or total ERK1/2 (1:1000; Cell Signaling Technologies, Danvers, MA, USA, cat# 4696) as previously described [96]. After washing with TBS-T, membranes were incubated with the appropriate diluted secondary antibody and were visualized using the Azure Imaging System (Dublin, CA, USA).

### 4.13. Migration Assay

RNCFs were plated on 2% gelatin-coated plates and allowed to adhere for 24 h. Cells were serum starved for 1 h followed by scraping the cells in a straight line using a P200 pipette tip to create a scratch. Cells were stained with fluorescein conjugated wheat germ agglutinin (WGA; 20 µg/mL; Sigma-Aldrich, St. Louis, MO, USA) to outline the cell and imaged over time on a Nikon Eclipse microscope at 10× magnification. The rate of closer was calculated by normalizing the area of the scratch to the original area using ImageJ (National Institutes of Health, Bethesda, MD, USA).

### 4.14. Statistical Analysis

Data presented are expressed as the mean ± SEM. Statistical analysis was performed using unpaired Student *t*-tests, one-way ANOVA with Tukey’s multiple comparison test, or two-way repeated-measures ANOVA where appropriate using Prism 5.0 software (GraphPad Software, San Diego, CA, USA) with *p* values indicated in the figure legends.

## Figures and Tables

**Table 1:** Transcript expressions of IL-1B, IL-6, IL-10, INFG and TNFA were examined by RT-qPCR in RNCFs treated with vehicle or isoproterenol. *n* = 7, *t* test, * *p* < 0.05 versus vehicle.

Gene Name	Vehicle	Isoproterenol
IL-1B (*n* = 7)	1.00 ± 0.10	0.96 ± 0.07
IL-6 (*n* = 7)	1.00 ± 0.26	2.33 ± 0.33 *
IL-10 (*n* = 7)	1.00 ± 0.28	1.58 ± 0.42
INFG (*n* = 7)	1.00 ± 0.25	1.14 ± 0.15
TNFA (*n* = 7)	1.00 ± 0.17	0.94 ± 0.14

## Supplementary Materials

Supplementary materials can be found at https://www.mdpi.com/1422-0067/21/22/8507/s1.

## Abbreviations

AMCF	Adult mouse cardiac fibroblast
βAR	β- adrenergic receptor
BM	Bone marrow
BMT	Bone marrow transplant
BrdU	Bromodeoxyuridine
cAMP	Cyclic adenosine monophosphate
Ctx	Cholera toxin
ECM	Extracellular matrix
IL	Interleukin
KO	Knockout
RNCF	Rat neonatal cardiac fibroblast
RT-qPCR	Reverse transcription-quantitative polymerase chain reaction
siRNA	Small interfering RNA
SOCS	Suppressors of cytokine signaling
