# Supplementary material for: β2-Adrenergic Receptors Increase Cardiac Fibroblast Proliferation Through the Gαs/ERK1/2-Dependent Secretion of Interleukin-6
Source: Int J Mol Sci. 2020 Nov 12;21(22):8507. doi: 10.3390/ijms21228507 (PMC7697911; doi:10.3390/ijms21228507)
Supplement: Supplementary file 1 [file ijms-21-08507-s001.pdf]

Supplemental Table S1. Primer sequences used for RT-qPCR.

Gene Name	Reference Sequence	Sequence
Rat ADRB1	NM_012701	Forward 5′-CAA GAC ACT GGG CAT CAT CA-3′<br>Reverse 5′-GCC AGT TGA AGA AGA CGA AGA-3′
Mouse ADRB2	NM_007419	Forward 5′-GCT GAT CTG GTC ATG GGA TT-3′<br>Reverse 5′-CGT CAC ACA CAG CAC ATC TA-3′
Rat ADRB2	NM_012492	Forward 5′-GAG CCT GCT GAC CAA GAA TAA-3′<br>Reverse 5′-GTG CAT CTG GAT AGG CAA GAA-3′
Mouse ADRB2	NM_007420	Forward 5′-AAG AAT AAG CCC GAG TGG T-3′<br>Reverse 5′-GTa GGC CTG GTT CGT GAA GA-3′
Rat IL1B	NM_031512	Forward 5′-CTA TGG CAA CTG TCC CTG AA-3′<br>Reverse 5′-GGC TTG GAA GCA ATC CTT AAT C-3′
Mouse IL6	NM_001314054	Forward 5′-CCT CCA TCC AGT TGC CTT CT-3′<br>Reverse 5′-CTC GA CTT GTG AAG TGG TAT AG-3′
Rat IL6	NM_012589	Forward 5′-GAA GTT AGA GTC ACA GAA GGA GTG-3′<br>Reverse 5′GTT TGC CGA GTA GAC CTC ATA G-3′
Rat IL10	NM_012854	Forward 3′-AGT GGA GCA GGT GAA GAA TG-3′<br>Reverse 3′-GAG TGT CAC GTA GGC TTC TAT G-3′
Rat INFG	NM_138880	Forward 3′-TGG ATG CTA TGG AAG GAA AGA G-3′<br>Reverse 3′-GAC TTC AAA GAG TCT GAG GTA GAA-3′
Rat TNFA	NM_012675	Forward 3′-ACC TTA TCT ACT CCC AGG TTC T-3′<br>Reverse 3′-GGC TGA CTT TCT CCT GGT ATG-3′
Mouse TPT-1	NM_009429	Forward 5′-ATC ATC TAC CGG GAC CTC ATC-3′<br>Reverse 5′-CCC TCT GTT CTA CTG ACC ATC T-3′
Rat TPT-1	NM_053867	Forward 5′-CTG CTG CTT ACC ATC CAT CA-3′<br>Reverse 5′-ACA ATG CCT CCA CTC CAA ATA-3′

Supplemental Figure 1

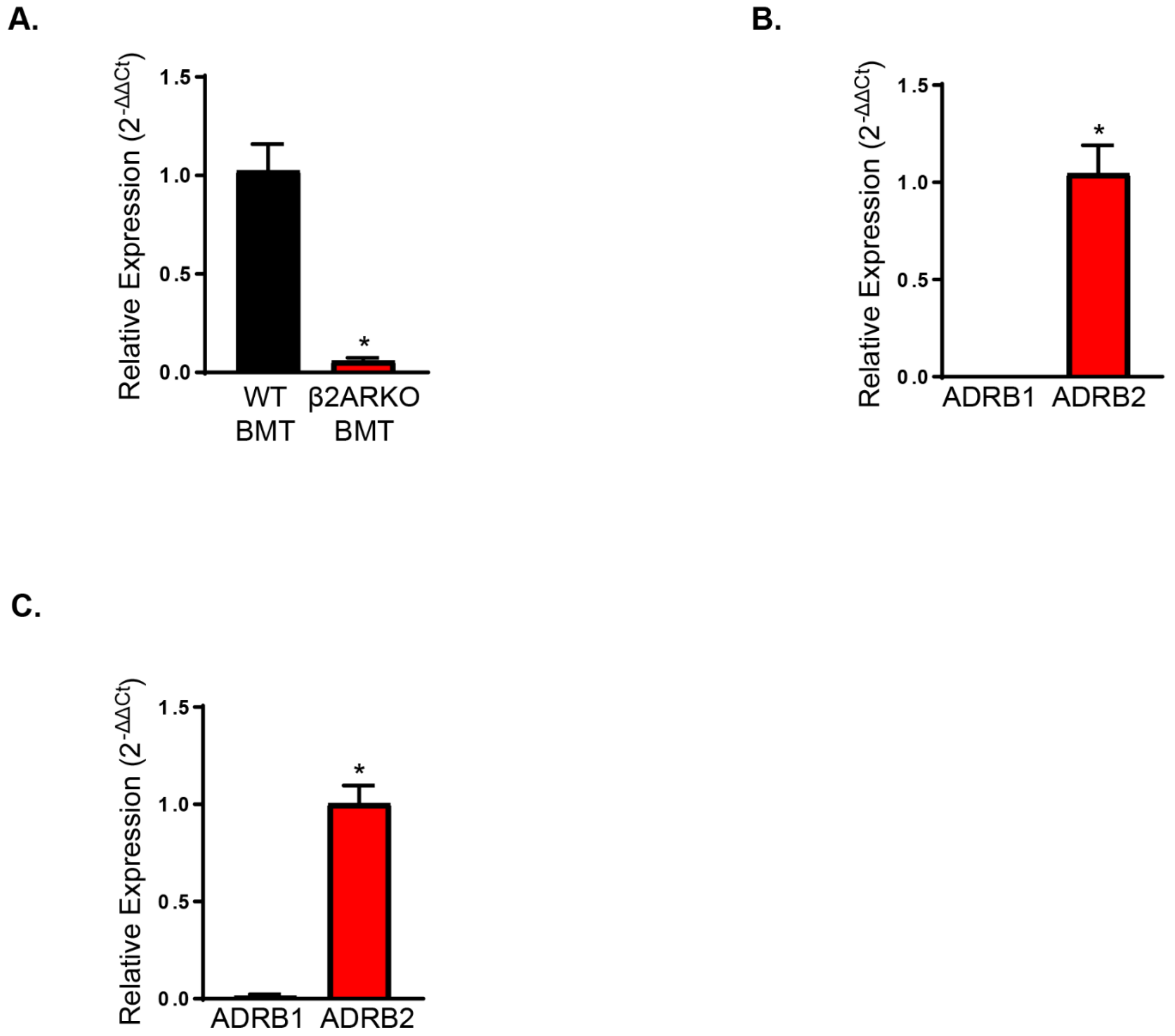

Supplemental Figure S1. βAR subtype expression. **A.** ADRB2 expression was quantified in reconstituted BM from WT and β2ARKO BMT mice by RT-qPCR. Expression is shown relative to WT BMT. n=4, t test, * $p < 0.05$. **B.** ADRB1 and ADRB2 expression was quantified in RNCF using RT-qPCR. Expression is shown relative to ADRB2 expression. n=6, t test, * $p < 0.05$ versus ADRB1. **C.** RT-qPCR was used to measure ADRB1 and ADRB2 expression in AMCF. Expression is shown relative to ADRB2 expression. n=, t test, * $p < 0.05$ versus ADRB1.

Supplemental Figure 2

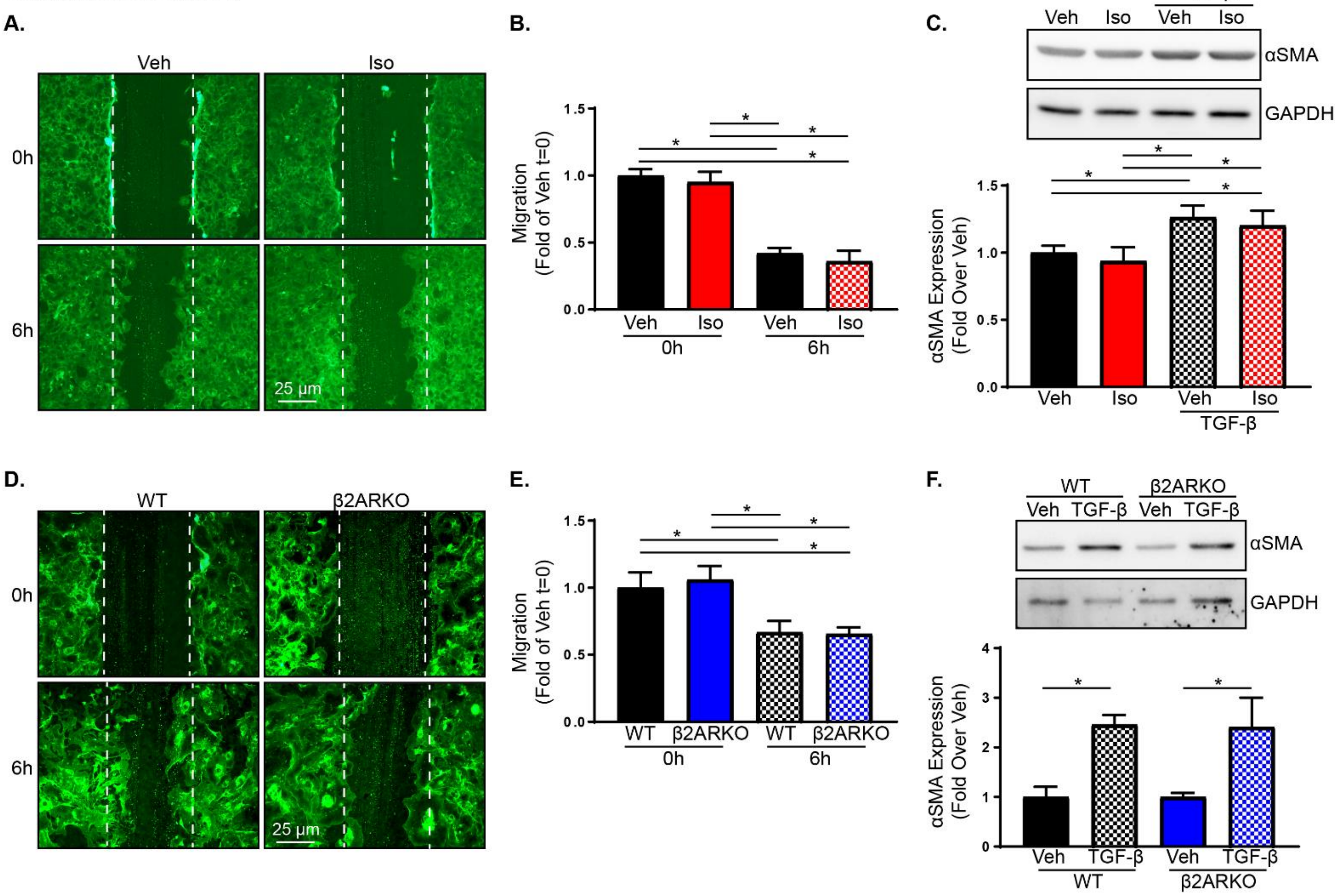

Supplemental Figure S2. The impact of β2AR on fibroblast functions. **A.** Representative scratch assay images from time=0 and 6h post-scratch RNCF treated with vehicle or isoproterenol. **B.** Quantified scratch assay data from vehicle and isoproterenol treated RNCF. Scratch area is normalized to vehicle treated time=0 RNCF. n=6, Two-Way ANOVA, * $p < 0.05$. **C.** α-smooth muscle actin immunoblot in RNCF treated with TGF-β in the presence or absence of isoproterenol. Expression is normalized to GAPDH and expressed as fold over vehicle. n=6, One-Way ANOVA, * $p < 0.05$. **D.** Representative scratch assay images from time=0 and 6h post-scratch in WT and β2ARKO AMCF. **E.** Quantified scratch assay data from WT and β2ARKO AMCF. Scratch area is normalized to WT time=0 AMCF. n=3, Two-Way ANOVA, * $p < 0.05$. **F.** Immunoblot for α-smooth muscle actin expression in WT and β2ARKO AMCF treated with vehicle or TGF-β. Expression is normalized to GAPDH and expressed as fold over WT vehicle. n=3, One-Way ANOVA, * $p < 0.05$.

Supplemental Figure 3

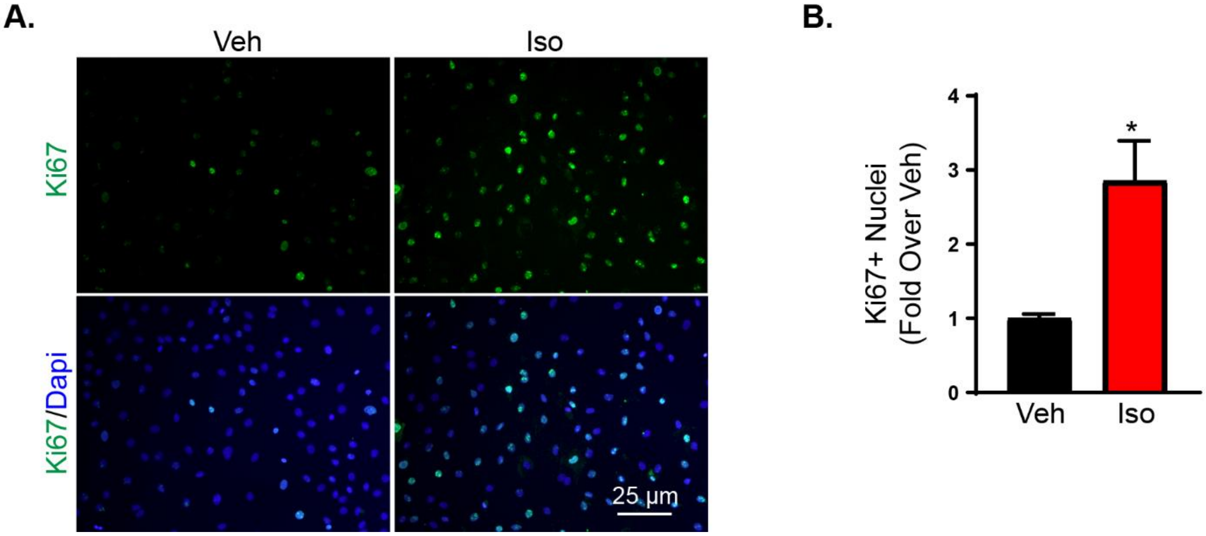

Supplemental Figure S3. β2AR increases fibroblast proliferation. **A.** Representative Ki67 (green, upper row) immunocytochemistry of RNCF treated with vehicle or isoproterenol. Ki67 merged with DAPI (blue) is shown in the lower panel. **B.** Quantification of Ki67-positive cells from vehicle and isoproterenol treated RNCF. n=8, t test, * $p < 0.05$.

Supplemental Figure 4

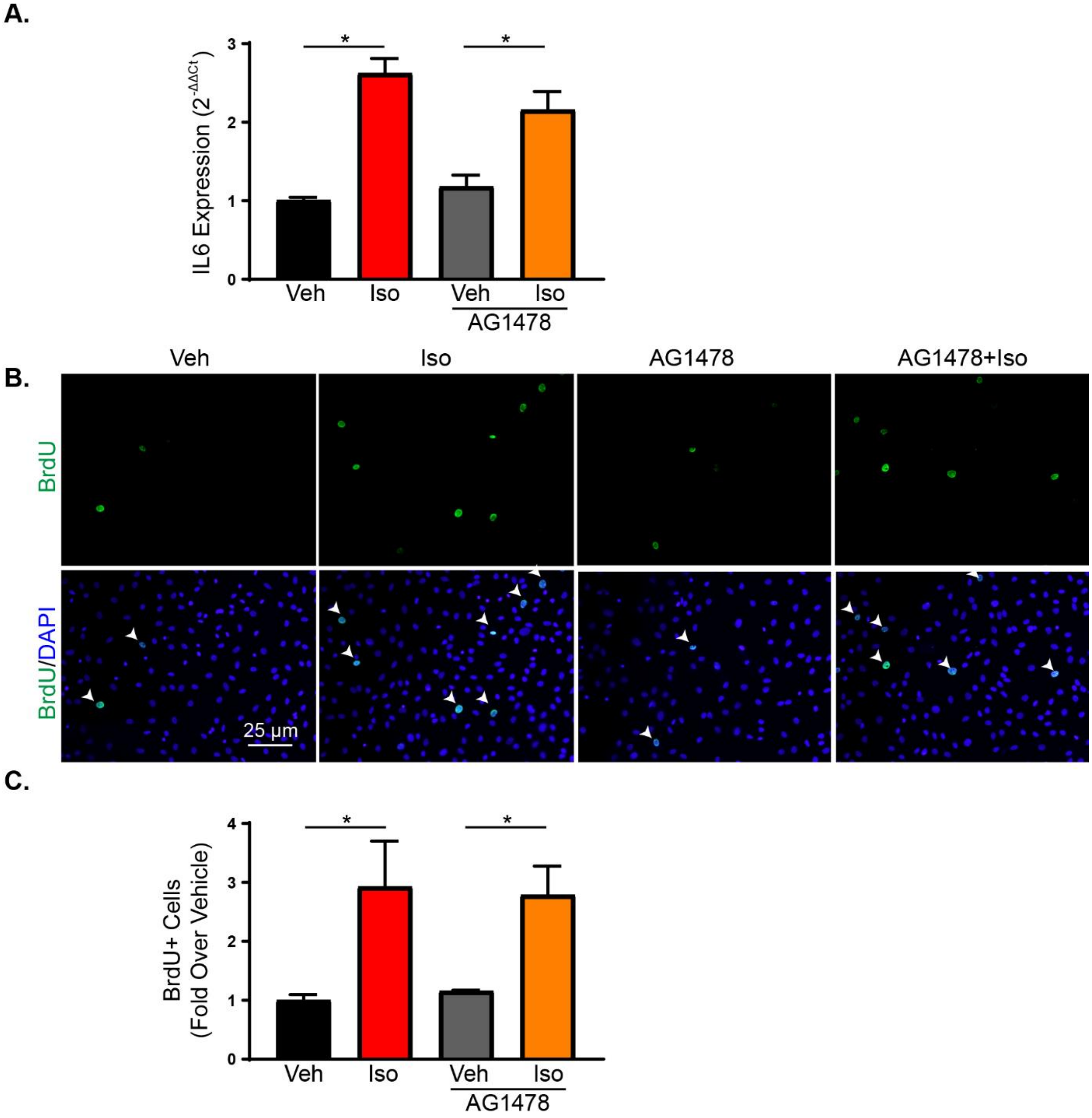

Supplemental Figure S4. β2AR-mediated G protein-independent signaling in cardiac fibroblasts. **A.** IL6 transcript expression was measured in RNCF treated with vehicle or isoproterenol in the presence or absence of AG1478. n=6, One-Way ANOVA, * $p < 0.05$. **B.** Representative BrdU (green) staining alone (top) or merged with DAPI (bottom) and quantification of BrdU-positive cells **(C)** from RNCF treated with vehicle or isoproterenol with or without pre-incubation with AG1478. Arrows indicate BrdU-positive nuclei. n=3, One-Way ANOVA, * $p < 0.05$.

Supplemental Figure 5

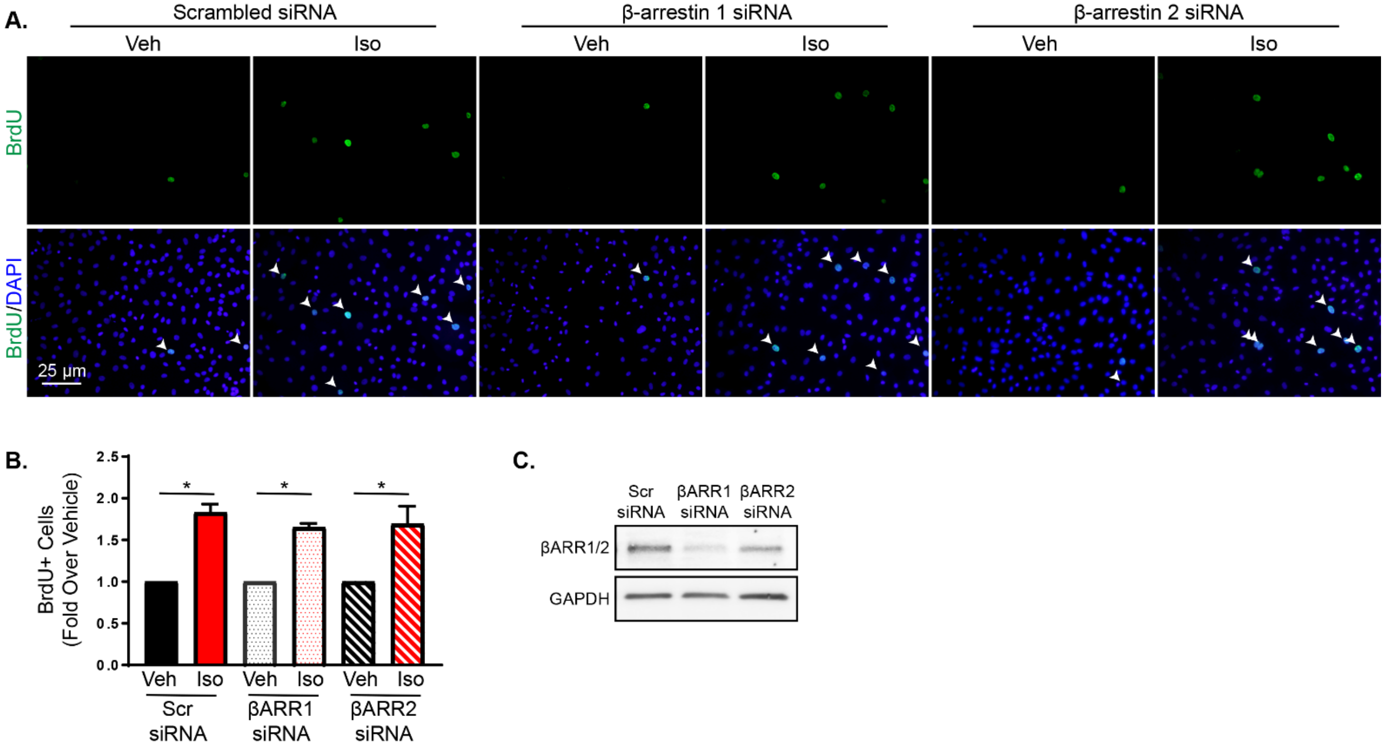

Supplemental Figure S5. β-Arrestin involvement in isoproterenol-mediated proliferation. **A.** Representative BrdU (green) staining alone (top) or merged with DAPI (bottom) and quantification of BrdU-positive cells **(B)** from scrambled, β-arrestin 1 or β-arrestin 2 siRNA transfected RNCF treated with vehicle or isoproterenol. Arrows indicate BrdU-positive nuclei. n=4, One-Way ANOVA, * $p < 0.05$. **C.** Immunoblot confirmation of β-arrestin 1 and 2 knockdown with scrambled, β-arrestin 1 or β-arrestin 2 siRNA transfection. GAPDH is shown as a loading control.

Supplemental Figure 6

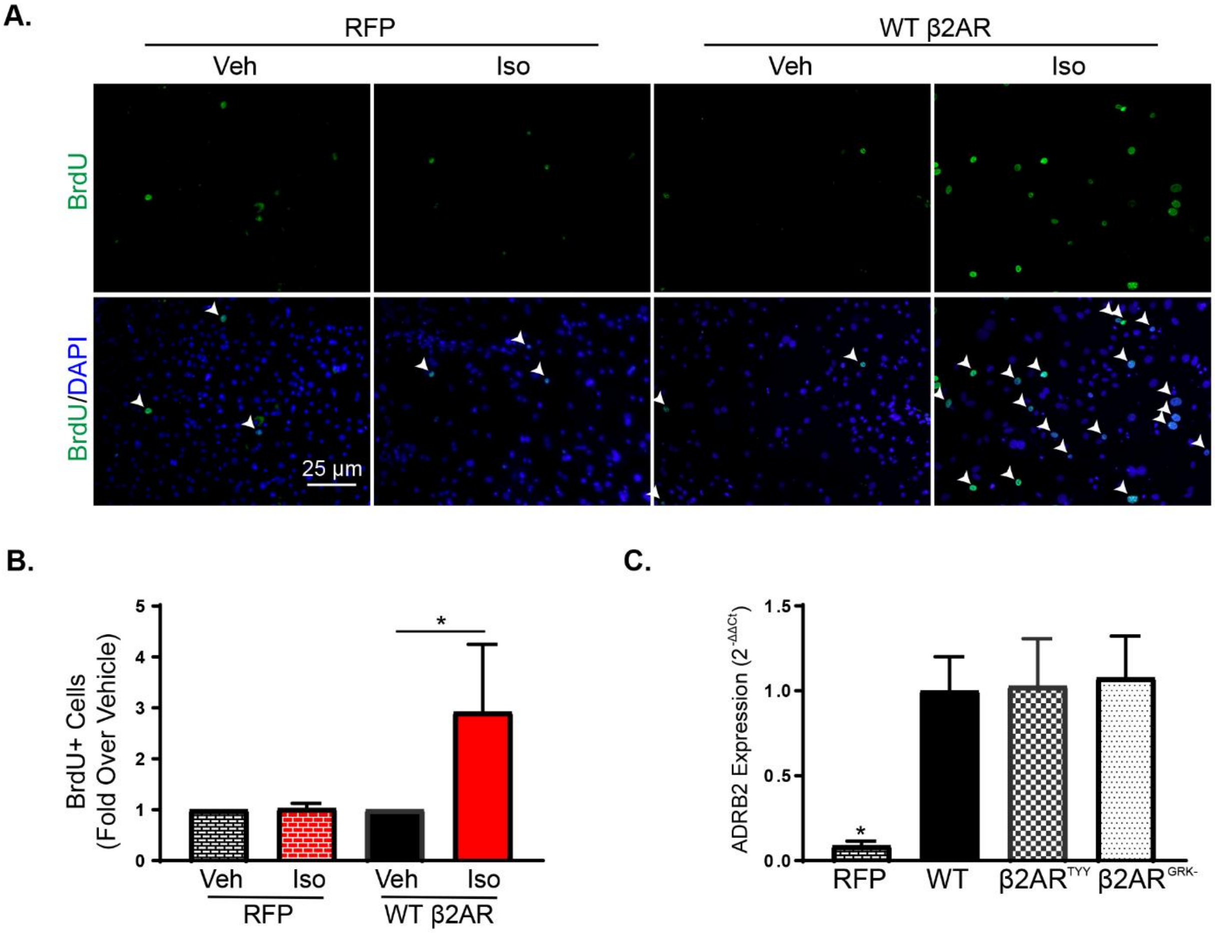

Supplemental Figure S6. β2AR-mediated G protein-dependent and independent signaling in fibroblast proliferation. **A.** Representative BrdU (green) staining alone (top) or merged with DAPI (bottom) and quantification of BrdU-positive nuclei **(B)** from vehicle and isoproterenol treated β2ARKO AMCF that were transduced with WT β2AR or RFP control lentivirus. Arrows indicate BrdU-positive nuclei. n=6, One-Way ANOVA, * $p < 0.05$. **C.** ADRB2 expression was quantified by RT-qPCR in β2ARKO AMCF that were transduced with lentivirus for WT β2AR, $\beta 2AR^{TYY}$, $\beta 2AR^{GRK-}$ or RFP control virus. n=6, One-Way ANOVA, * $p < 0.05$ versus WT β2AR.

Supplemental Figure 7

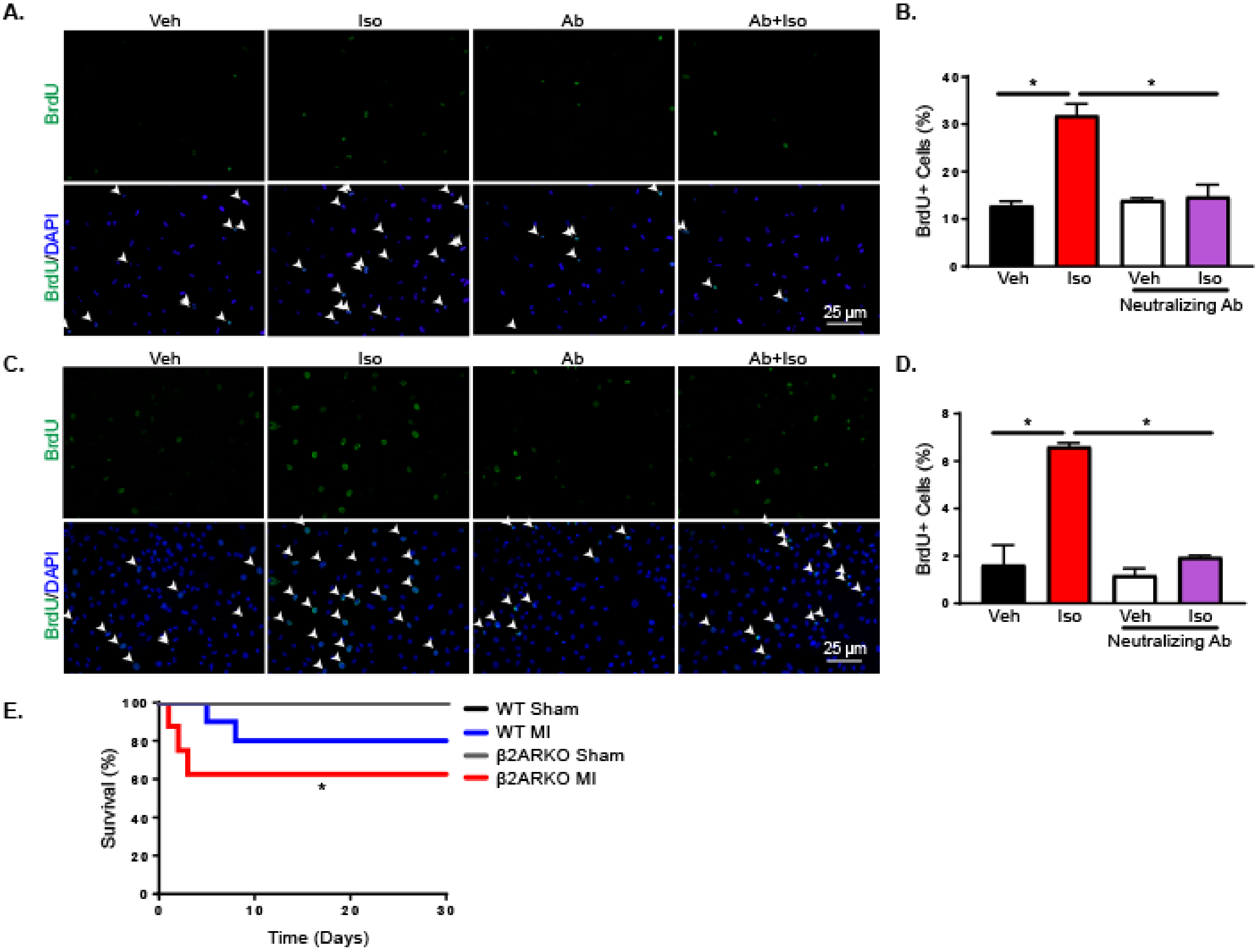

Supplemental Figure S7. Dermal fibroblasts were isolated from WT mice and treated with vehicle or isoproterenol in the presence or absence of an IL-6 neutralizing antibody. **A.** Representative BrdU (green) staining alone (top) or merged with DAPI (bottom) and quantification of BrdU-positive nuclei **(B)** from vehicle and isoproterenol treated dermal fibroblasts. Arrows indicate BrdU-positive nuclei. n=3, One-Way ANOVA, * $p < 0.05$. **C.** Representative BrdU (green) staining alone (top) or merged with DAPI (bottom) from mouse embryonic fibroblasts treated with vehicle or isoproterenol with our without IL-6 neutralizing antibody pretreatment. Arrows indicate BrdU-positive nuclei. **D.** Quantification of mouse embryonic fibroblast BrdU staining. n=4, One-Way ANOVA, * $p < 0.05$. **E.** Survival over time in WT and β2ARKO mice following sham or myocardial infarction surgery. n=10. Log-rank, * $p < 0.05$.
